# Polysialic acid sustains cancer cell survival and migratory capacity in a hypoxic environment

**DOI:** 10.1038/srep33026

**Published:** 2016-09-09

**Authors:** Sara M. Elkashef, Simon J. Allison, Maria Sadiq, Haneen A. Basheer, Goreti Ribeiro Morais, Paul M. Loadman, Klaus Pors, Robert A. Falconer

**Affiliations:** 1 Institute of Cancer Therapeutics, Faculty of Life Sciences, University of Bradford, West Yorkshire BD7 1DP, U.K; 2 Department of Pharmacy, School of Applied Sciences, University of Huddersfield, Huddersfield HD1 3DH, U.K

## Abstract

Polysialic acid (polySia) is a unique carbohydrate polymer expressed on the surface of NCAM (neuronal cell adhesion molecule) in a number of cancers where it modulates cell-cell and cell-matrix adhesion, migration, invasion and metastasis and is strongly associated with poor clinical prognosis. We have carried out the first investigation into the effect of polySia expression on the behaviour of cancer cells in hypoxia, a key source of chemoresistance in tumours. The role of polysialylation and associated tumour cell migration and cell adhesion were studied in hypoxia, along with effects on cell survival and the potential role of HIF-1. Our findings provide the first evidence that polySia expression sustains migratory capacity and is associated with tumour cell survival in hypoxia. Initial mechanistic studies indicate a potential role for HIF-1 in sustaining polySia-mediated migratory capacity, but not cell survival. These data add to the growing body of evidence pointing to a crucial role for the polysialyltransferases (polySTs) in neuroendocrine tumour progression and provide the first evidence to suggest that polySia is associated with an aggressive phenotype in tumour hypoxia. These results have significant potential implications for polyST inhibition as an anti-metastatic therapeutic strategy and for targeting hypoxic cancer cells.

Polysialic acid (polySia) is an α-2,8-glycosidically linked polymer of sialic acid, and a developmentally regulated post-translational modification of NCAM (neuronal cell adhesion molecule)^1^. Cancers of neuroendocrine-origin exhibit selective high level expression of polySia-NCAM as part of the tumour glycocalyx, a term used to describe the myriad of functionally-important carbohydrates that are to be found on the surface of cancer cells^2^. Tumours where polySia expression has been identified notably include neuroblastoma^3^,^4^, lung cancer^5^,^6^ and many others^1^,^7^,^8^,^9^,^10^,^11^. Crucially, whilst high levels are expressed during embryonic development, peripheral adult organs do not express polySia-NCAM. This means that the polysialyltransferase (polyST) enzymes (ST8SiaII and ST8SiaIV) responsible for polySia biosynthesis^12^ have received considerable interest as novel anti-metastatic drug targets, particularly ST8SiaII, which is thought to be the prominent enzyme in tumours^1^.

PolySia-NCAM expression strongly correlates with the migration and invasion of tumour cells^13^ and with aggressive, metastatic disease and poor clinical prognosis in the clinic^1^. Its detailed roles in tumour growth and dissemination continue to emerge, but involve disruption of homo- and heterophilic NCAM interactions, and in modulation of key intracellular signalling pathways, notably FGFR-1, ERK1/2, FAK and c-MET/ALK^1^,^14^,^15^. Furthermore, it has long been proposed that polySia-NCAM expression may protect the tumour cell from immunosurveillance mechanisms, in a manner analogous to bacteria expressing polySia^16^ and that it is closely associated with tumour chemoresistance^17^.

The tumour microenvironment is intimately connected with the evolution of cancers and the limited success of cancer treatments. Hypoxia, a condition of low oxygen tension occurring in poorly vascularised areas of tumours, has profound effects on cancer cell growth^18^,^19^, metastasis^20^,^21^, susceptibility to apoptosis^22^,^23^ and resistance to radiotherapy and chemotherapy^24^,^25^. Within solid tumours, oxygen delivery to neoplastic and stromal cells in different regions of the tumour varies considerably due to the chaotic nature of the tumour vasculature and the diffusion limit of oxygen of just a few hundred micrometres. Oxygen gradients exist across the tumour with decreasing levels of oxygen as distance from a blood vessel increases. Whilst different levels of hypoxia are thus likely to exist in different parts of the tumour, in general, hypoxic cancer cells are associated with a more aggressive, invasive phenotype^26^,^27^,^28^. The altered glycosylation of cancer cells appears to play a key role in this; promoting loss of cell-cell adhesion and cell migration^29^,^30^. However, how glycosylation changes under hypoxia and what effect, if any, this has on the behaviour of cancer cells, such as their growth, survival and invasive potential remain largely unexplored. Given the key role played by polySia in neuroendocrine tumour progression, we hypothesised that polySia may play a crucial role in tumour cell behaviour under hypoxic conditions.

## Materials and Methods

### Cell lines

Human neuroblastoma SH-SY5Y (ATCC^®^ CRL2266™) and DLD-1 colorectal adenocarcinoma (ATCC^®^ CCL221™) cell lines were obtained from the American Type Culture Collection (ATCC). Human neuroblastoma SH-SY5Y cells were maintained in MEM medium and nutrient mixture F-12 Ham (1:1), supplemented with 10% foetal bovine serum, 1% sodium pyruvate and 1% glutamine. DLD-1 colorectal adenocarcinoma cell lines were maintained in RPMI media supplemented with 10% foetal bovine serum, 1% sodium pyruvate and 1% glutamine. C6-STX and C6-WT cells were obtained from the Fukuda group, Sanford-Burnham Prebys Medical Discovery Institute, La Jolla, CA, USA (for full details, see Suzuki *et al.*)^31^. Briefly, C6-STX glioma cells were prepared by transfecting wild-type C6 cells (C6-WT) with the pcDNA3-STX plasmid inserted with cDNA encoded full length human ST8SiaII (also known as STX)^31^. Both cell lines were maintained in minimum essential medium (MEM Alpha Eagle) with ultraglutamine I, deoxyribonucleoside and ribonucleosides supplemented with 10% foetal bovine serum. All cell lines were maintained in a humid atmosphere of 5% CO_2_ and 95% air at 37 °C.

For studies carried out under conditions of low oxygen tension (“hypoxic conditions”), all media used was incubated for at least 48 hours under hypoxic conditions (0.1% oxygen) before being added to the cells.

### MTT cell viability assay

C6 glioma cells (5 × 10^3^ cells/well) were seeded in 96-well plates. After overnight attachment, the cells were treated with different concentrations of cobalt chloride (Sigma–Aldrich) to induce pseudohypoxia. Following 24 h treatment, cells were washed and fresh media was added. Cells were incubated for another 72 hours and MTT (0.5 mg/ml) was then added followed by incubation for a further 4 hours. The MTT metabolic product (formazan) was dissolved in DMSO (200 μL), and its absorbance was measured at 560 nm. Cell survival was calculated in relation to control (vehicle-treated) cells.

### *In vitro* cell migration assay

Effects on tumour cell migration were analysed using a simple 2D scratch assay^13^,^32^. Cells were seeded into six-well plates at different concentrations (1 × 10^6^ cells for SH-SY5Y, 0.5 × 10^6^ cells for C6 and 0.8 × 10^6^ cells for DLD-1), and plates were then incubated overnight at 37 °C in a 5% CO_2_ humidified atmosphere. Once a confluent monolayer had formed, a 200 μl pipette tip was used to create a scratch wound. The monolayer was then washed with growth medium (1 ml) to remove floating cells and replaced with fresh medium (2 ml) containing only 2% FBS to limit cell proliferation. For experiments under hypoxia, the scratch was performed in a hypoxia station (Whitley H35 hypoxystation; Don Whitley, UK) at 0.1% O_2_ and hypoxia-equilibrated media was used. Images of the scratch at the start of the experiment were acquired using a Lumascope 500 microscope (Etaluma, USA) and reference points were marked to obtain the same field during the image acquisition after the incubation.

The plates were incubated in either normoxic (i.e. normal laboratory conditions) or hypoxic conditions for 16, 24 and 41 hours for C6, SH-SY5Y and DLD-1 cells respectively. After incubation, plates were placed under the Lumascope 500 microscope, reference points were matched, the photographed region acquired at the first image was aligned and a second image was acquired.

The images acquired were analysed quantitatively by using Image J software. For each image, distances between one side of scratch and the other were measured at certain intervals (μm). By comparing the images from time 0 to the last time point the % wound closure was calculated.

### Chromatographic cell-based analysis of polySia expression

C6 cells were seeded at the density of 2 × 10^5^ cells/well in a six-well plate and incubated at 37 °C in either hypoxic or normoxic conditions. After 48 hours, cells were collected by trypsinisation followed by centrifugation for 5 minutes at 1000 rpm. Using routine mild acidic hydrolysis for polySia solubilisation^33^,^34^, cell pellets were treated with 100 μL of 0.5% Igepal CA-630 (in distilled water) followed by incubation at 37 °C for 0.5–1 h and vortex mixing for 5 minutes. The insoluble material was removed by centrifugation and the supernatant was used for analysis. For Endo-N treatment for polySia solubilisation, cells were treated with Endo-N (3 μg/ml) for 24 hours prior collecting the media. One well was used as negative control where no Endo-N was added.

For determination of polySia content, samples were hydrolysed in 0.1 M trifluoroacetic acid (TFA) for 2 h at 80 °C. TFA was removed under vacuum and the resulting sialic acid residues were incubated with the DMB reagent for 24 h at 4 °C as described previously^13^.

### Mass spectrometric analysis of DMB-sialic acid

DMB-sialic acid was subjected to analysis by electrospray UPLC-MS using an Acquity UPLC Waters system and a Quattro Premier XE (Waters corporation, USA) mass spectrometer and an Acquity UPLC BEH C18 column (2.1 × 100 mm). The mobile phases used were: ammonium formate (5 mM; mobile phase A) and methanol (mobile phase B), with a flow rate of 0.3 ml/min and isocratic method of 20% mobile phase B. The peaks and masses were integrated using Masslynx software. The source temperature was set to 120 °C. MRM transitions acquired at unit resolution in both the Q1 and Q3 quadrupoles to maximise specificity. Cone voltage (CV) was adjusted to 25 V while collision energy (CE) was adjusted to 20 V using positive ionisation mode. The transitions detected are summarised in Table 1.

### Cell-cell adhesion assay

C6 cells were incubated under hypoxic or normoxic conditions for 48 hours at 37 °C. Matrigel^©^ (200 μl, 40 μg/ml) (VWR, USA) solution was added to each well of a 24-well plate and incubated at 37 °C. After two hours, excess Matrigel was removed and the wells were washed twice with cold PBS. Blocking solution (DMEM media with %10 FBS, 500 μl) was added to each well. The plates were then incubated at 37 °C. After 30 minutes the wells were washed once with cold PBS and fresh culture medium (200 μl) was added and the plates were incubated at 37 °C.

2 × 10^4^ cells in 100 μl medium were added to the pre-coated 24-well plate. Culture medium (300 μl) was added to one well to be used as a blank to calculate the background staining. The plates were incubated at 37 °C for 30 minutes. Non-adherent cells were removed by inverting the plates on a plastic reservoir. Wells were washed two times with 300 μl ice-cold PBS containing 1 mM CaCl_2_ and 1 mM MgCl_2_. Cells were fixed by adding ice-cold 100% methanol (300 μl). After 10 minutes, wells were washed with PBS three times and of 0.5% crystal violet solution, prepared in 20% ethanol (100 μl), was added. Plates were placed on a shaker and incubated with crystal violet solution for 10 minutes at room temperature. Excess crystal violet solution was then removed. Crystal violet was then recovered by adding 200 μl of 100% methanol to each well. 100 μl of the extracted crystal violet was transferred to 96-well plate. Absorbance of the samples was measured spectrophotometrically at a wavelength of 540 nm. The background absorbance of multi-well plates was also measured and subtracted from the 540 nm measurements.

Absorbance readings acquired for each sample were analysed quantitatively. Background absorbance of the multi-well plates was subtracted and the % cell adhesion relative to C6-WT cells was calculated (% adhesion = mean absorbance of the C6-WT cells /mean absorbance of C6-STX cells in the same condition × 100). Three independent experiments were carried out, each comprising 4 technical replicates.

### Trypan blue exclusion assay for cell viability

C6 cells were seeded in T75 flasks with seeding density of 2.5 × 10^4^ cells/flask, with one flask per treatment incubated in normoxic conditions and the other in hypoxic (0.1% O_2_) conditions for 96 hours. 0.4% solution of trypan blue was prepared in PBS solution (pH 7.2) and 0.1 mL of trypan blue stock solution was added to 1 mL of trypsinised cells. Each haemocytometer chamber was loaded with 10 μl of the solution and examined immediately under a microscope at low magnification. The number of trypan blue-stained cells and the number of total cells was counted. The % trypan blue-stained cells was calculated as follows: % dead cells = (Average number of trypan blue-stained cells count ÷ average total cell count) × 100.% cell viability was also calculated as follows: % Viable cells = [1.00 – (Number of blue cells ÷ Number of total cells)] × 100.

### Flow cytometry (Annexin V apoptosis detection assay)

C6 cells were seeded (8 × 10^5^ cells) in T75 flasks under normoxic or hypoxic (0.1% O_2_) conditions at 37 °C. After incubation for 96 h, media was collected and cells were trypsinised. Trypsinised cells, along with the collected media, were then centrifuged at 1000 g for 5 minutes. After centrifugation, media was discarded and cell pellets were re-suspended in HEPES buffer (1 ml) as provided with the Annexin V_FLUOS staining kit (Roche) in accordance with the manufacturer’s instructions. Cells were further centrifuged (1500 g, 5 minutes) and pellets were re-suspended in 100 μl HEPES buffer containing Propidium iodide and Annexin V as previously described^35^. After 15 minutes, 0.5 ml of Annexin-binding buffer was added and cell samples were analysed by flow cytometry.

### Western blot analysis

For CoCl_2_ treatment, C6-STX and C6-WT cells were seeded at a density of 2 × 10^5^ cells/well in a six-well plate and incubated for 24 hours at 37 °C in a 5% CO_2_ humidified atmosphere to allow cells to adhere. After 24 hours, medium was removed and fresh medium containing different concentrations of CoCl_2_ was added. Two wells were used as a control where no CoCl_2_ was added. After 16 hours cells were collected.

Samples were resolved on 12% polyacrylamide gels and blotted onto PVDF membranes (Amersham). Non-specific antibody binding was blocked via incubation with skimmed milk (0.05 g/ml) and the blot was probed with rat anti-HIF-1α antibody (R&D systems, UK) (1:100 dilution) or rat anti-LDH-A antibody (Abcam, UK) (1:500 dilution) overnight at 4 °C. Antibody reactivity was detected by horseradish peroxidase (HRP)-conjugated antibody and chemiluminescence using ECL-Plus (Amersham).

### RT-PCR analysis

#### RNA purification and quantification

C6 were incubated in normoxic or hypoxic conditions for 48 h, after which RNA was extracted using the RNeasy Mini Kit (Qiagen, UK) according to the manufacturer’s instructions. The concentration and quality of the eluted RNA were determined by measuring absorbance at 260 nm and 280 nm using a NanodropTM2000 (Thermo Fisher Scientific) spectrophotometer and determining the 260/280 ratio.

2000 ng of total RNA was reverse transcribed into single-stranded cDNA using the High Capacity cDNA Reverse Transcription Kit (Life technologies) according to manufacturer’s handbook. Once the reaction finished, samples were stored at −20 °C.

#### Real-time quantitative PCR

qPCR was carried out using the TaqMan Universal PCR Master Mix (Applied Biosystems). The PCR reaction consisted of 12.5 μl of 2X master mix, 1.25 μl of 20X TaqMan Gene Expression Assay Mix, and 11.25 μl cDNA diluted in dH2O. The cDNA samples were diluted 1:5 and 2 μl of diluted cDNA was used per reaction. A 96-well MicroAmp Optical plate (Applied Biosystems) was used and all reactions were performed in triplicate. The validated Taqman primers (Applied Biosystems) utilised are listed in Table 2. qPCR reactions were performed using a AB7500 Real time PCR system (Applied Biosystems). The fold-change in gene expression was determined using the 2-ddCT method^36^.

### Statistics

A minimum of three independent biological experiments were performed for all studies. Statistical analyses were performed using Excel software. Differences between two groups were evaluated with Student’s t test (Two tailed).

## Results and Discussion

### Evaluation of polySia expression in C6-STX cells under hypoxic conditions

Cells were initially evaluated to assess the effect of hypoxia on polySia expression. The isogenic C6 glioma cells served as an ideal model in this regard. First reported by the Fukuda group^31^, C6-STX cells have been engineered to express ST8SiaII and thus express polysialylated NCAM (polySia-NCAM)^13^. The merits of these cells as a model for polySia-expressing cancer cells are well established^13^,^31^,^37^. C6-STX cells migrate and invade significantly faster than C6-WT (wild type) cells *in vitro*^13^,^31^ and *in vivo*^31^. Our own experiments have additionally confirmed that levels of polySia expression in C6-STX cells are similar to those observed in naturally expressing cell lines (e.g. SH-SY5Y, data not shown). C6-STX and C6-WT cells are otherwise genetically identical (expressing NCAM, the acceptor for polySia), only differing in the expression of ST8SiaII and associated polySia^31^. We were thus able to reasonably attribute any effects observed in the subsequent experiments to the presence of polySia.

C6-STX cells were incubated at 37 **°**C in either 0.1% oxygen, 5% carbon dioxide and 95% nitrogen (hypoxia) or 5% carbon dioxide and 95% air (normoxia). After 48 h, cells were harvested and polySia expression was quantified using a cell-based chromatographic assay (see Methods).

**Figure 1:**
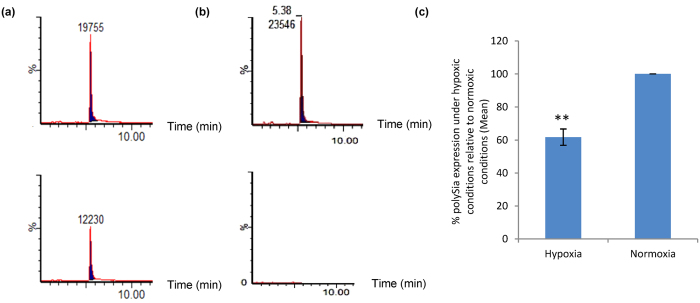
Effect of hypoxia (48 hours, 0.1% O_2_) on polySia expression in C6-STX cells. (**a**) Mild acidic hydrolysis combined with UPLC/MS/MS analysis used to detect polySia in C6-STX cells under normoxia (upper trace) and hypoxia (lower trace, after 48 hours). Y-axis represents % abundance of polySia, x-axis represents time. (**b**) Elution standard for standard DMB-labelled sialic acid (upper trace) and negative control sample where no Endo-N was added as described in the material and methods section (lower trace). (**c**) Quantification of the mean polySia levels as determined by HPLC. (**P ≤ 0.01).

Interestingly, a comparison of the polySia expression of C6-STX cells under hypoxia and normoxia revealed a marked difference. C6-STX cells exhibited a significant reduction in polySia expression of polySia under hypoxia conditions (61.8 ± 4.8% of that measured under normoxia, P = 0.01, Fig. 1). The use of C6-WT cells as a negative control confirmed a lack of polySia expression under both conditions (data not shown) and validated the assay given the isogenic nature of the cells. Cell-based quantification of cell-surface polySia was carried out using both endoneuraminidase-N (Endo-N, an enzyme that selectively cleaves polySia) and mild acidic hydrolysis for the removal of polySia from the cell surface, two techniques routinely available to us in our laboratory. Similar results were obtained from both experiments, giving confidence in the result obtained (SD ± 10%).

These data show that steady-state levels of exogenously expressed polySia are reduced in C6-STX cells under hypoxic conditions. These findings are exciting and represent the first such evidence that hypoxia affects polySia expression in tumour cells. That said, these results are consistent with previous studies that have more broadly suggested that acute hypoxia produces a degree of glycocalyx degradation^38^.

### Effect of hypoxia on polySia-mediated tumour cell migration

PolySia has been shown to play a key role in tumour cell migration. Generally speaking, there are many reports demonstrating that hypoxia modulates cancer cell migration^39^,^40^. Given our determination that polySia levels were reduced under hypoxic conditions, implications for tumour cell migration were investigated.

**Figure 2:**
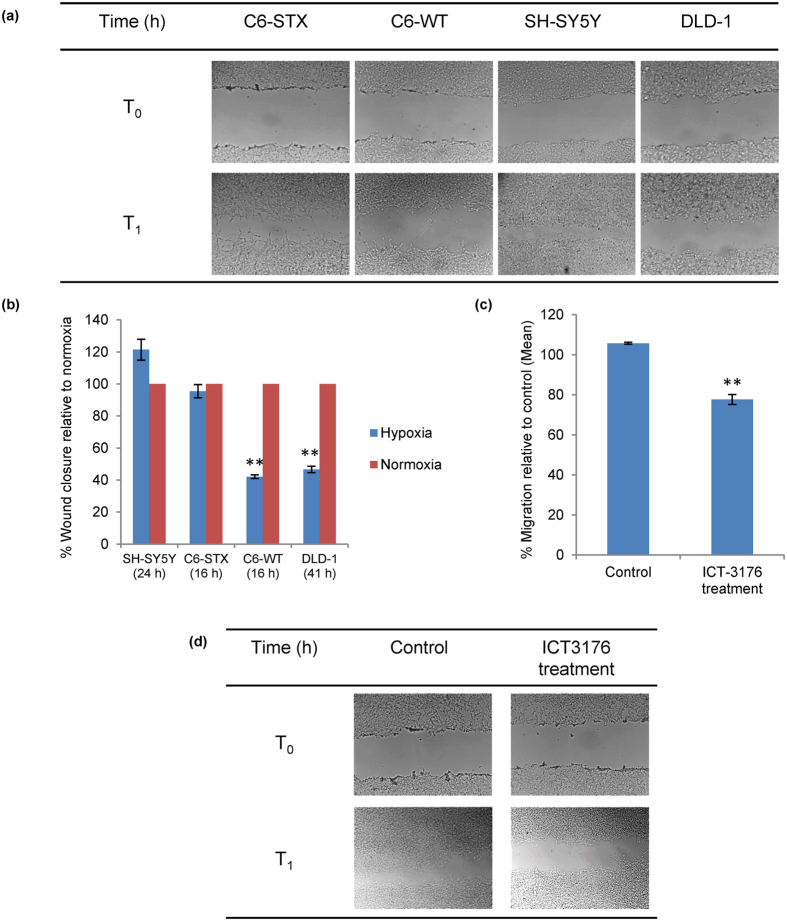
Effect of polySia on the migration of tumour cells under hypoxic conditions. (**a**) Representative images of the migration of SH-SY5Y, C6-STX cells (expressing polySia), and control cell lines C6-WT and DLD-1 using time-lapse microscopy under hypoxia, showing the wound size immediately and 16, 24 and 41 hours after scratching; (**b**) Graph representing the quantification of wound closure calculated by measuring the reduction of wound area over time, using Image J software. Wound closure under hypoxia is calculated as a percentage of wound closure under normoxia for each cell line; (**c**) Effect of ICT-3176 treatment on C6-STX cell migration under hypoxia; (**d**) Representative images of the migration of C6-STX cells after treatment with ICT-3176 compared to control untreated cells under hypoxia. T_1_ = 16 h (C6-STX, C6-WT); 24 h (SH-SY5Y); 41 h (DLD-1). (**P ≤ 0.01).

The effect of polySia expression on tumour cell migration under hypoxic conditions was once again analysed using the C6 glioma cell line pair: C6-STX (polySia^+^/NCAM^+^) and C6-WT (polySia^−^/NCAM^+^). We, along with our collaborators before us, have previously demonstrated that C6-STX cells migrate significantly faster than C6-WT cells in normoxia^13^,^31^. We additionally evaluated naturally polySia-expressing neuroblastoma cell line SH-SY5Y (polySia^+^/NCAM^+^), and utilised DLD-1 colorectal cancer cells as a negative control (polySia^−^/NCAM^−^). Migration was evaluated using a simple scratch assay^32^, routinely used in our laboratory. For the two polySia-expressing cell lines (C6-STX and SH-SY5Y), no difference in cell migration was observed between the hypoxic and normoxic conditions. This is intriguing given our finding that polySia expression is reduced in C6-STX cells under hypoxia. Interestingly, the control cells (C6-WT and DLD-1), which do not express polySia, showed a significant and marked reduction in cell migration under hypoxia (Fig. 2a,b).

Given these results, we hypothesised that a polyST inhibitor would reduce polySia expression further and thus may reduce migration of polySia-expressing cells under hypoxia. We have previously demonstrated that polyST inhibitor ICT-3176 reduces tumour cell migration in polySia-expressing cells (including C6-STX and SH-SY5Y cells) with high selectivity^41^. C6-STX cells were thus treated with ICT-3176 (250 μM) under hypoxic conditions. Cell migration was compared with non-treated cells. Treatment of the cells with ICT-3176 resulted in significant inhibition of C6-STX cell migration (22.4 ± 2.5%, Fig. 2c,d) in hypoxia. As expected, ICT-3176 treatment showed no effect on C6-WT cells.

Taken together, these results demonstrate that the polySia-expressing tumour cells evaluated under hypoxia are able to maintain the migratory capacity observed in normoxia, and this is despite the significant reduction in polySia expression levels noted earlier. Furthermore, polyST inhibition results in reduced migration in hypoxia, as it does in normoxia.

### Effect of hypoxia on polySia-mediated cancer cell-matrix adhesion

It was previously reported that polySia modulates cancer cell adhesion through modulation of NCAM-NCAM interactions and NCAM interaction with other molecules in addition to exerting a repulsive force created by the negative charge on the polySia chains. Furthermore, evidence has been reported suggesting that polySia and hypoxia modulate the c-MET signalling pathway, which has a significant role on cancer cell adhesion^42^.

In order to investigate the effect of hypoxia on polySia-mediated tumour cell-matrix adhesion, an assay was performed with C6-STX and C6-WT cells after 48 hour growth in either normoxic or hypoxic conditions. C6-STX cells were less able to adhere to Matrigel^©^ than C6-WT cells, irrespective of oxygen conditions. Adhesion capacity in polySia-expressing C6-STX cells was approximately 15% less than that observed in control C6-WT cells. There was no difference, however, in cell adhesiveness between normoxic and hypoxic conditions for either C6-STX or C6-WT cells (P = 0.9 for C6-STX cell adhesion in hypoxia compared to normoxia; P = 0.3 for C6-WT adhesion in hypoxia compared to normoxia, see Supplementary data S1).

These results suggest that unlike migration, hypoxia does not appear to have any significant effect on polySia-mediated cell-matrix adhesion, at least to Matrigel in this case. The lack of a difference between adhesion observed in hypoxia and normoxia is perhaps surprising given the lower quantity of polySia expressed by C6-STX cells under hypoxic conditions. It may be that polySia modulates cell adhesion by interacting with different signalling pathways under hypoxic conditions, such as c-MET which is over expressed in hypoxic conditions^43^,^44^. This is interesting and certainly worthy of further exploration.

### Effect of polySia expression on cancer cell survival in hypoxia

**Figure 3:**
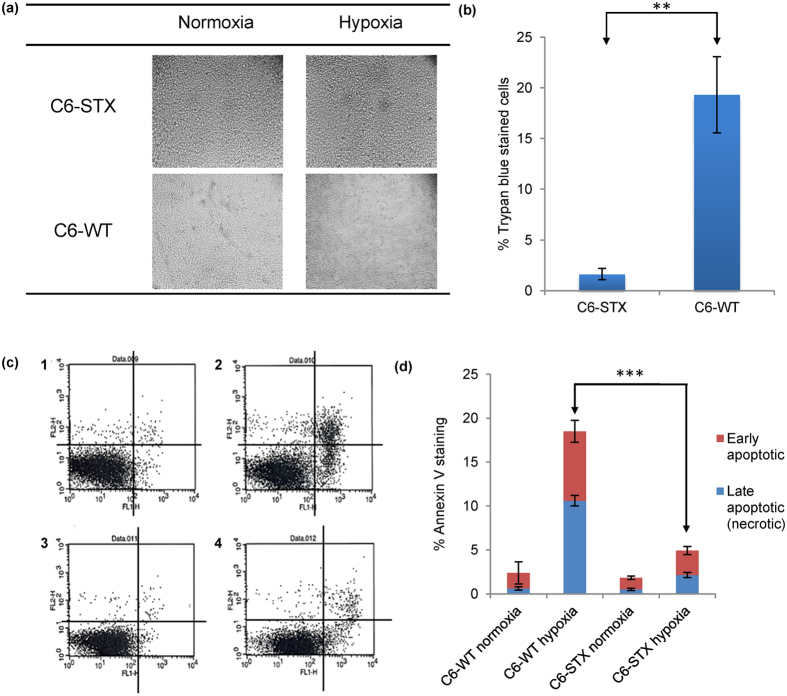
Effect of polySia expression on the survival of cancer cells in hypoxia. (**a**) Morphological change in C6-STX and C6-WT cells after incubation under hypoxic or normoxic conditions for 96 h; (**b**) Trypan blue exclusion assay, showing the effect of hypoxia on the viability of C6 cells. The graph represents the mean of three independent experiments; (**c**) Flow cytometry analysis of C6-STX and C6-WT cells apoptosis under hypoxic and normoxic conditions after 96 h. The x-axis shows levels of annexin V cellular staining whereas levels of propidium iodide staining are shown on the y-axis; lower left quadrant of dot plots 1–4: live cells (annexin V-negative, propidium iodide-negative); lower right quadrant: early apoptotic cells (annexin V-positive, propidium iodide-negative); upper right quadrant: late apoptotic or necrotic cells (annexin V-positive, propidium iodide-positive); upper left quadrant: mechanically damaged cells (annexin V-negative, propidium iodide-positive). C1 represents C6-WT in normoxia, C2 represents C6-WT cells in hypoxia, C3 represents C6-STX cells in normoxia, C4 represents C6-STX cells in hypoxia; (**d**) Percentage of annexin V staining of cell lines under each condition. (**P ≤ 0.01; ***P ≤ 0.001).

We next considered whether polySia plays any role in cell survival under hypoxic conditions. In order to determine this, C6-STX and C6-WT cells were subjected to hypoxia for 96 h while control cells were incubated for the same duration under normoxic conditions. After 96 h, cells were examined under the microscope and were subsequently collected. Viable and dead cell counts were determined using trypan blue staining. Interestingly, polySia-expressing cells were associated with significantly higher cancer cell viability under hypoxic conditions compared to controls (Fig. 3a). The trypan blue exclusion assay revealed that after 96 h under hypoxia, C6-WT control cells demonstrated significantly reduced viability, as evidenced by a greater number of trypan blue-stained cells (Fig. 3b). C6-WT cells demonstrated cell viability of 80% (i.e. 19.7 ± 3.8% trypan blue-stained cells, Fig. 3b) compared to C6-STX polySia-expressing cells with cell viability of 98% (i.e. 1.6 ± 0.6% trypan-blue stained cells, Fig. 3b).

In order to further analyse this reduction in cell survival observed under hypoxia and potential mechanisms of increased cell death in the C6-WT cells, levels of apoptosis in hypoxia-cultured C6-STX and C6-WT cells were determined. As shown in Fig. 3c, the apoptotic fraction of C6-WT cells was significantly higher than that of C6-STX cells under hypoxic conditions (18.5% apoptotic cells in C6-WT compared to 4.9% apoptotic cells in C6-STX, i.e. 73.5% less apoptosis in C6-STX cells, Fig. 3d, Table 3), while no significant difference was observed in normoxia. These results strongly suggest that polySia expression plays a key role in enhancing C6 glioma cell survival in hypoxia.

For comparison, we additionally performed this experiment using SH-SY5Y cells, which naturally express polySia. The levels of apoptosis in SH-SY5Y cells cultured in hypoxia and normoxia were similarly compared. No significant difference in the percentage of viable cells cultured in hypoxia or normoxia was identified, mirroring the results obtained using C6-STX cells (Supplementary data S2).

### Investigating a potential role of hypoxia-inducible factor-1 (HIF-1) in the altered behaviour of polySia-expressing tumour cells under hypoxic conditions

A critical mediator of the hypoxic response is the transcription factor hypoxia-inducible factor 1 (HIF-1) that upregulates expression of proteins that promote angiogenesis, anaerobic metabolism, metastasis and many other survival pathways^45^. Glucose metabolism is upregulated by many cancer cells under hypoxia, generating lactate through the enzyme lactate dehydrogenase A (LDH-A)^46^, an established HIF-1a target gene^47^,^48^. HIF-1 activity in tumours depends on the availability of the HIF-1α subunit, the levels of which increase under hypoxic conditions and through the activation of oncogenes and/or inactivation of tumour suppressor genes^49^,^50^.

In order to investigate whether HIF-1α plays any role in the altered polySia-mediated cancer cell behaviour observed under hypoxic conditions, we utilised an established experimental tool to investigate HIF-1 dependency of hypoxia-induced phenomena, namely cobalt chloride (CoCl_2_). CoCl_2_ administration under normoxia is thus a simple means by which to mimic the HIF-1-dependent processes that normally occur under hypoxia. CoCl_2_ stabilises HIF-1α by blocking post-translational modification by prolyl hydroxylase^51^,^52^. Whereas CoCl_2_ lacks exquisite selectivity for HIF-1α, it is routinely used in this context as a tool prior to more complex experiments^53^,^54^. In the next few experiments, the effect of HIF-1α induction using CoCl_2_ on the key polySia-induced behavioural changes of cancer cells under hypoxia were examined.

### Determination of CoCl_2_ cytotoxicity

In order to determine the most suitable CoCl_2_ concentrations for studying the effect of HIF1α induction on both migration and survival of C6 cell lines, an MTT viability assay was performed.

**Figure 4:**
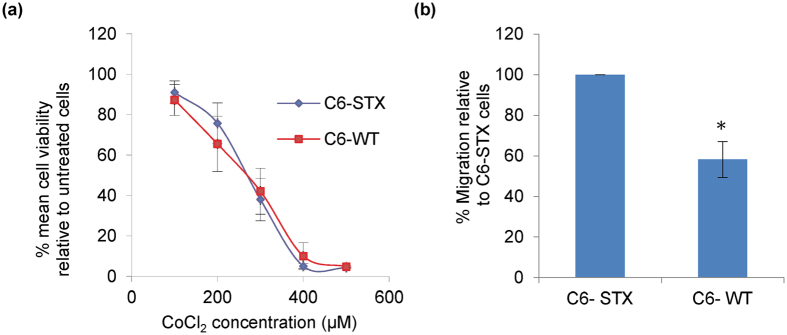
Effect of CoCl_2_ treatment on C6 cell lines. (**a**) Determination of IC_50_ of CoCl_2_ in C6-STX and C6-WT cell lines; (**b**) Quantification of migration of C6-STX and C6-WT cells after treatment with CoCl_2_, calculated by measuring the reduction of wound area over time using Image J software. The percentage of C6-WT wound closure after CoCl_2_ treatment is calculated as a percentage of the C6-STX wound closure. (*P ≤ 0.05).

In this experiment, the viability of C6-STX and C6-WT cells was examined after incubation with different concentrations of CoCl_2_ for 24 hours to determine IC_50_ values. These were determined to be 258 ± 10 μM for C6-STX cells and 289 ± 12 μM and C6-WT cells (Fig. 4a). For the migration assay, a concentration of CoCl_2_ that did not cause a significant reduction in the viability of either cell line was used, i.e. 100 μM. For studying the ability of cells to survive under stress conditions, 200 μM of CoCl_2_ was utilised, a concentration causing approximately 20% loss in viability of cell population.

In the absence of being able to reliably detect changes in HIF-1α expression directly, we focused instead on induction of LDH-A expression to confirm effects of CoCl_2_, which are well established. Whilst important in itself, LDH-A induction by CoCl_2_ has additionally been routinely utilised by others as an indirect indicative marker of transcriptionally active and functional HIF-1a expression, given its strong correlation with HIF-1α expression^55^,^56^. Expression of LDH-A in both C6-STX and C6-WT CoCl_2_-treated cells was significantly higher than control untreated cells. On this basis, we could additionally assume induction of HIF-1α expression and pseudohypoxia (Supplementary data S4).

### Effect of CoCl_2_ on polySia-mediated tumour cell migration

In order to investigate whether the effect of polySia expression on enhancing migration of cancer cells under hypoxic conditions is HIF-1α-related, the migration of C6-STX and C6-WT cells was examined following treatment with 100 μM CoCl_2_ (a non-toxic concentration, as determined above). Both C6-STX and C6-WT cells exhibited a significant reduction in tumour cell migration following CoCl_2_ treatment (Supplementary data S3). However, CoCl_2_ had a greater effect on C6-WT cells than C6-STX cells (35 ± 15% greater migration in C6-STX cells compared with C6-WT cells, P = 0.02, Fig. 4b). When considered in conjunction with the results obtained under hypoxia (Fig. 2a), this suggests a link between polySia-mediated migration of tumour cells under hypoxic conditions and LDH-A induction, with implications for HIF-1α.

### Effect of CoCl_2_ on polySia-expressing cancer cell survival

Following our observation the polySia-expressing tumour cells exhibit enhanced cell survival under hypoxia, we evaluated the effect of CoCl_2_ on cell survival. Percentage cell survival of C6-STX and C6-WT cells was examined after incubation with CoCl_2_ (200 μM; a concentration selected to study the ability of cells to survive under stress conditions) using the trypan blue exclusion assay. In contrast to the results obtained under hypoxia, no significant difference in cell survival was observed between C6-STX and C6-WT cells (P = 0.26, Supplementary data S5). This suggests that the enhancement of cell survival induced by polySia expression under hypoxic conditions may not be correlated to HIF-1α upregulation.

### Differential expression of selected HIF-1α induced genes in C6-STX versus C6-WT cells under hypoxia

Possible cross-talk between polySia and glucose transporters has been previously suggested: *N*-Acetyl-D-mannosamine (ManNAc) and *N*-acetyl-D-glucosamine (GlcNAc) are the essential precursors of *N*-acetylneuraminic acid (NeuAc), the precursor monomer of polySia. Studies on the specificity of the ManNAc transport system have revealed that addition to the uptake mixture of glucose, fructose, mannose, glucosamine, mannosamine and GlcNAc (5 mM), but not other sugars, caused a marked inhibition in ManNAc transport (70, 60, 70, 60, 65 and 85%, respectively)^57^,^58^. Although this effect was observed in *E. coli*, given the link between hypoxia and increased glucose metabolism in cancer cells, this raised the question of possible cross-talk between polySia and these processes. In initial studies, we selected three key glucose metabolism regulators, namely glucose transporter 1 (GLUT-1), hexokinase 2 (HK-2) and the aforementioned LDH-A^55^ for evaluation^59^,^60^,^61^. The change in expression of these genes was measured under hypoxia and normalised to expression under normoxia.

**Figure 5:**
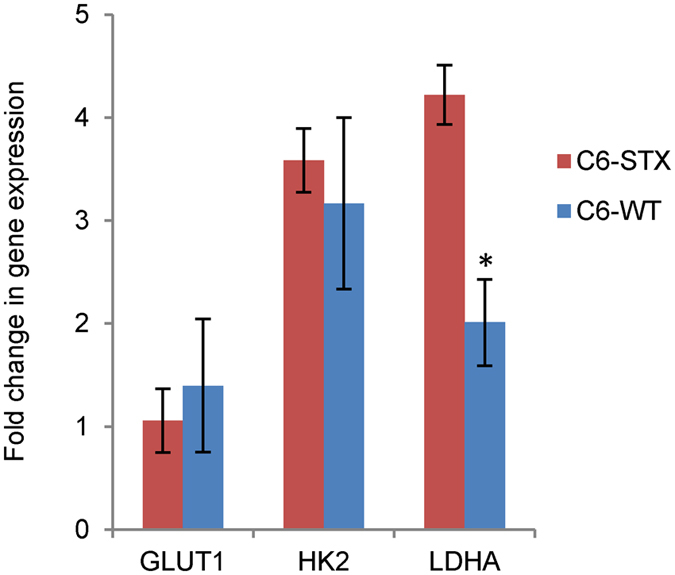
Fold-change in gene expression of selected hypoxia target genes under hypoxic conditions in C6-STX compared to C6-WT cells. (*P ≤ 0.05).

Gene analysis of the C6 isogenic cells revealed a significant increase in LDH-A gene expression in C6-STX cells under hypoxic conditions as compared to WT cells. This is a significant finding, suggesting a possible role for LDH-A in polySia-mediated changes in cancer cell behaviour in hypoxia (Fig. 5). LDH-A has previously been reported to promote cancer cell survival in multiple cancer cell lines^35^,^46^,^62^, and has also been shown to promote cancer cell migration under hypoxic conditions^63^.

## Conclusion

The role of polySia in neuroendocrine tumours in tumour cell growth, dissemination and metastasis has now been widely reported. Whilst polySia expression has previously been proposed to be associated with chemoresistance, the significance of polySia expression in a hypoxic environment has not been studied. We have shown that while hypoxia leads to a reduction in polySia expression, this is strongly associated with increased cell survival, and sustains a migratory capacity. Initial mechanistic studies indicate a potential role for HIF-1 in sustaining polySia-mediated migratory capacity, but not cell survival. Furthermore, our study additionally provides the first evidence of a role for LDH-A, a key regulator of glucose metabolism under hypoxia in cancer cells, in these processes.

This study paves the way for more detailed studies in a broader array of neuroendocrine cancer cell lines, and invites further investigations using more complex *in vitro* models (e.g. 3D spheroids) and ultimately *in vivo*. Our ability to modulate polySia expression using small molecule polyST inhibitor ICT-3176, in conjunction with our striking finding that cells engineered to express polySia exhibit significantly enhanced survival in a hypoxic environment, raises exciting therapeutic implications for the targeting of hypoxic cells and for future anti-metastatic and combination therapies.

## Additional Information

**How to cite this article**: Elkashef, S. M. *et al.* Polysialic acid sustains cancer cell survival and migratory capacity in a hypoxic environment. *Sci. Rep.* **6**, 33026; doi: 10.1038/srep33026 (2016).

## Supplementary Material

Supplementary Information

## Figures and Tables

**Table 1:** Transition data for mass spectrometric analysis of DMB-sialic acid.

Transition	Molecular weight (MW)	Charge Z	Parent ion (m/z)	Daughter ion (m/z)	CV (V)	CE (eV)	Dwell time (sec)
1	425.4	+1	426	229.23	25	20	0.2
2	425.4	+1	426	313.02	25	20	0.2

**Table 2:** Validated Taqman primers utilised in PCR experiments.

Target gene (C6 cell lines)	TaqMan gene expression assay
GLUT1	Rn01402419_g1
HK2	Rn00562457_m1
LDH-A	Rn00820751_g1

**Table 3:** Summary percentages of annexin V-stained C6 cells under normoxia and hypoxia.

Cell line and conditions	Apoptotic cells (%)		Viable cells (%)
	Late apoptotic (necrotic)	Early apoptotic	
C6-WT (Normoxia)	0.64 ± 0.19	1.75 ± 1.24	96.22 ± 1.14
C6-WT (Hypoxia)	10.6 ± 0.60	7.89 ± 1.24	79.03 ± 1.50
C6-STX (Normoxia)	0.53 ± 0.13	1.33 ± 0.19	97.48 ± 0.37
C6-STX (Hypoxia)	2.16 ± 0.29	2.75 ± 0.46	94.45 ± 0.04
