# Supplementary material for: Polysialic acid sustains cancer cell survival and migratory capacity in a hypoxic environment
Source: Sci Rep. 2016 Sep 9;6:33026. doi: 10.1038/srep33026 (PMC5017143; doi:10.1038/srep33026)
Supplement: Supplementary Information [file srep33026-s1.pdf]

Polysialic acid sustains cancer cell survival and migratory capacity in a hypoxic environment

Sara M. Elkashef,[a] Simon Allison,[b] Maria Sadiq,[a] Haneen Basheer,[a] Goreti Ribeiro Morais,[a] Paul M. Loadman,[a] Klaus Pors[a] and Robert A. Falconer[a]

[a] Institute of Cancer Therapeutics, Faculty of Life Sciences, University of Bradford, West Yorkshire BD7 1DP, U.K. Tel: +44 (0)1274 235842; Email: r.a.falconer1@bradford.ac.uk

[b] Department of Pharmacy, School of Applied Sciences, University of Huddersfield, Huddersfield HD1 3DH, U.K.

Supplementary data 1 (S1)

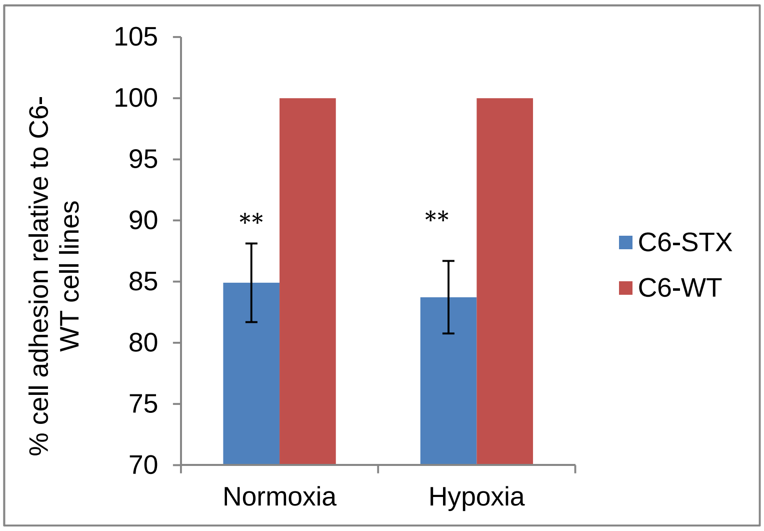

S1. Effect of hypoxia on the polySia-mediated adhesiveness of cancer cells. A polySia-mediated reduction in cell adhesion to Matrigel© in C6-STX but not C6-WT cell line was observed under both normoxia and hypoxia. (**P≤0.01)

Supplementary data 2 (S2)

Cell line and conditions	Apoptotic cells		Viable cells
	Late apoptotic (necrotic)	Early apoptotic	
SH-SY5Y (Normoxia)	6.16± 0.15	10.36 ± 2.01	81.94 ± 2.19
SH-SY5Y (Hypoxia)	4.41 ± 0.02	7.29 ± 0.29	85.54 ± 0.43

S2. **Percentage of annexin V staining of SH-SY5Y cells under hypoxic and normoxic conditions**

Supplementary data 3 (S3)

	**T0**	**T16**
**C6-STX, Control**	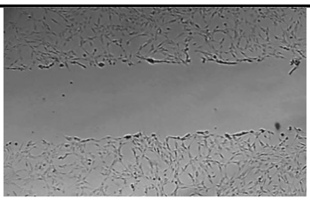	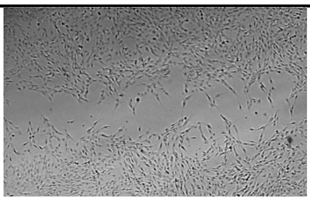
**C6-STX, $CoCl_2$, 130 µM**	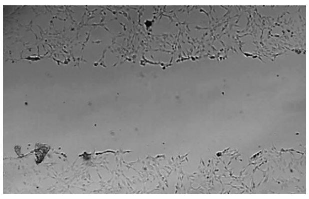	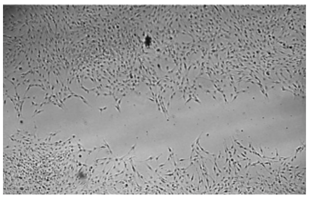
**C6-WT, Control**	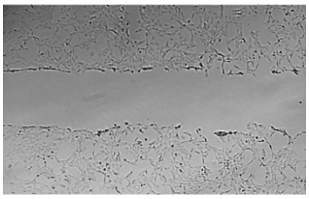	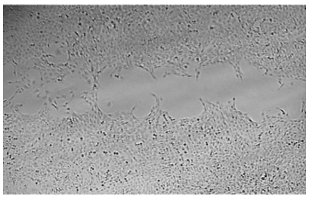
**C6-WT, $CoCl_2$, 130 µM**	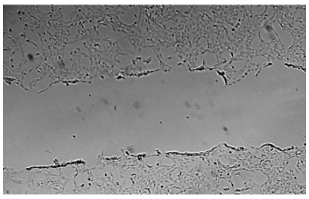	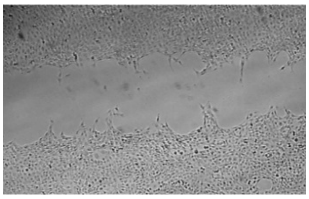

S3. **Migration of C6-STX and C6-WT cells following $CoCl_2$ treatment.** Wound size immediately and 16 hours after scratching illustrates wound closure of C6-STX and C6-WT cells with/without 100 µM $CoCl_2$.

Supplementary data 4 (S4)

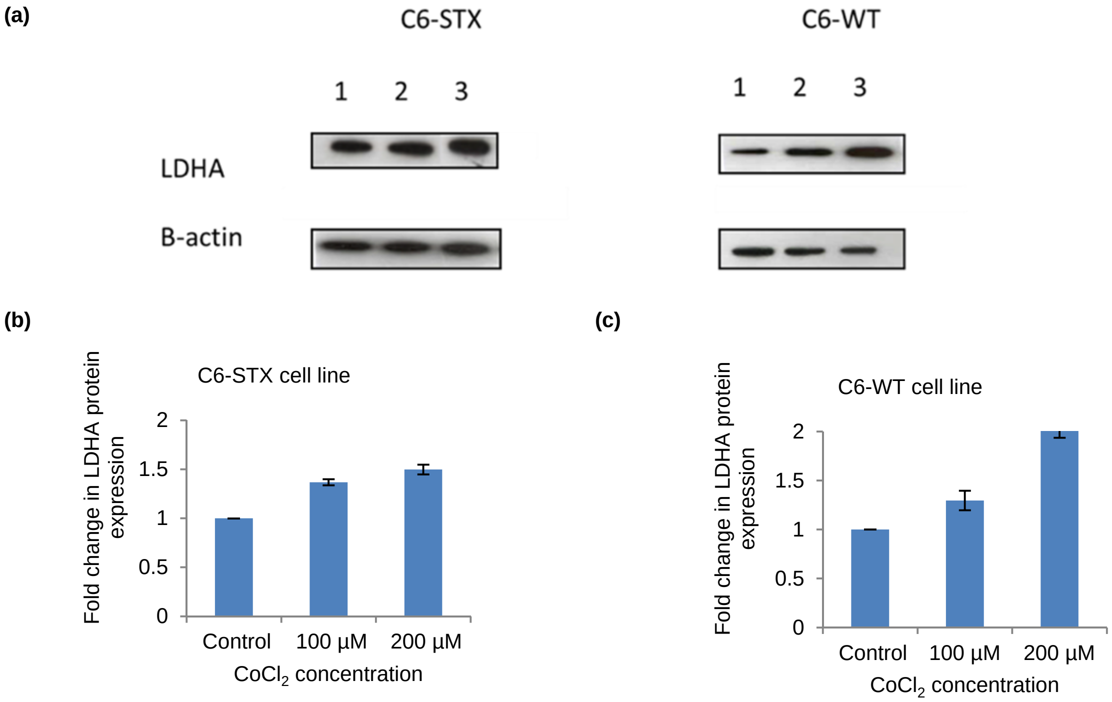

S4. Effect of $CoCl_2$ treatment on C6 cell lines. (A) Expression of LDHA after treatment of C6-STX and C6-WT cells with different concentrations of $CoCl_2$. Lane 1 represents control untreated cells, lane 2 after 130 µM $CoCl_2$ and lane 3 after 200 µM $CoCl_2$. Results are representative of two independent experiments; (B) quantification of fold-change in LDHA expression for C6-STX cells after treatment with 100 µM $CoCl_2$ (16 hours) and 200 µM $CoCl_2$ (24 hours); (C) quantification of fold-change in LDHA expression of C6-WT after treatment with 100 µM $CoCl_2$ (16 hours) and 200 µM $CoCl_2$ (24 hours).

Supplementary data 5 (S5)

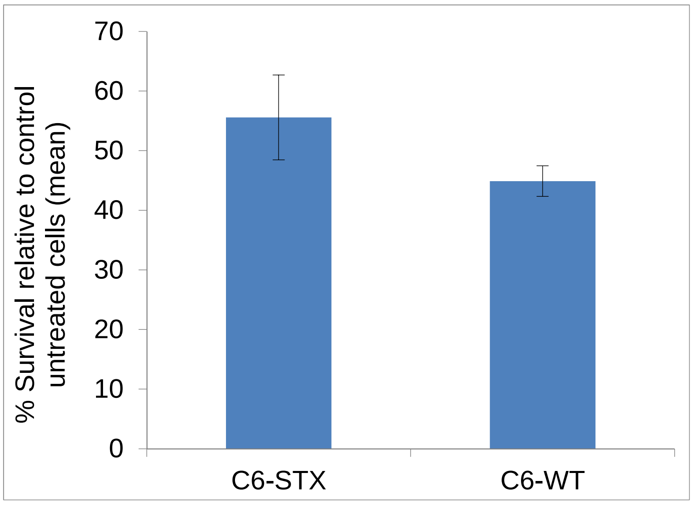

S5. Effect of HIF-1α induction on the survival of C6-STX and C6-WT cells. ($P \geq 0.05$, not significant)
